# Real-time colorectal polyp detection using a novel computer-aided detection system (CADe): a feasibility study

**DOI:** 10.1007/s00384-022-04258-9

**Published:** 2022-09-27

**Authors:** E. Soons, T. Rath, Y. Hazewinkel, W. A. van Dop, D. Esposito, P. A. Testoni, P. D. Siersema

**Affiliations:** 1 Department of Gastroenterology and Hepatology, Radboud Institute for Health Sciences, Radboud University Medical Center, 9101, 6500 HB Nijmegen, the Netherlands; 2 Department of Internal Medicine 1, Division of Gastroenterology, Friedrich-Alexander-University, Ludwig Demling Endoscopy Center of Excellence, Erlangen Nuernberg, Germany; 3 Gastroenterology and Gastrointestinal Endoscopy Unit, Vita-Salute San Raffaele University, Scientific Institute San Raffaele, Milan, Italy

**Keywords:** Endoscopy, Computer-aided detection (CADe) system, Colorectal polyp, Colorectal adenoma, Detection

## Abstract

**Background and aims:**

Colonoscopy aims to early detect and remove precancerous colorectal polyps, thereby preventing development of colorectal cancer (CRC). Recently, computer-aided detection (CADe) systems have been developed to assist endoscopists in polyp detection during colonoscopy. The aim of this study was to investigate feasibility and safety of a novel CADe system during real-time colonoscopy in three European tertiary referral centers.

**Methods:**

Ninety patients undergoing colonoscopy assisted by a real-time CADe system (DISCOVERY; Pentax Medical, Tokyo, Japan) were prospectively included. The CADe system was turned on only at withdrawal, and its output was displayed on secondary monitor. To study feasibility, inspection time, polyp detection rate (PDR), adenoma detection rate (ADR), sessile serrated lesion (SSL) detection rate (SDR), and the number of false positives were recorded. To study safety, (severe) adverse events ((S)AEs) were collected. Additionally, user friendliness was rated from 1 (worst) to 10 (best) by endoscopists.

**Results:**

Mean inspection time was 10.8 ± 4.3 min, while PDR was 55.6%, ADR 28.9%, and SDR 11.1%. The CADe system users estimated that < 20 false positives occurred in 81 colonoscopy procedures (90%). No (S)AEs related to the CADe system were observed during the 30-day follow-up period. User friendliness was rated as good, with a median score of 8/10.

**Conclusion:**

Colonoscopy with this novel CADe system in a real-time setting was feasible and safe. Although PDR and SDR were high compared to previous studies with other CADe systems, future randomized controlled trials are needed to confirm these detection rates. The high SDR is of particular interest since interval CRC has been suggested to develop frequently through the serrated neoplasia pathway.

**Clinical Trial Registration:**

The study was registered in the Dutch Trial Register (reference number: NL8788).

**Supplementary Information:**

The online version contains supplementary material available at 10.1007/s00384-022-04258-9.

## Introduction

Colorectal cancer (CRC) is the third most common and second most lethal cancer worldwide [1]. CRC typically develops from benign, precancerous lesions. Approximately 80–85% of CRCs develop from adenomas following the adenoma-carcinoma pathway. An additional 15–20% of CRCs develop through the serrated neoplasia pathway, of which sessile serrated lesions (SSLs) are considered the most important precursor lesions [2, 3].

Although colonoscopy is the most accurate screening modality for detecting and removing colorectal polyps, a substantial number of polyps is still missed. Earlier studies have reported a polyp miss rate (PMR) of 21–28%, an adenoma miss rate (AMR) of 22–26%, and a SSL miss rate of 27% [4, 5]. As expected, miss rates are higher when polyp size is smaller; for adenomas > 10 mm, the AMR is 2–6%, while for diminutive polyps (1–5 mm), it is reported to be as high as 26–28% [4, 5]. Missed lesions have a risk of developing into CRC, and it is thought that at least 50% of all interval cancers (iCRCs; defined as CRC diagnosed between screening and post-screening surveillance colonoscopies) develop from missed lesions during colonoscopy [6]. Interestingly, CRCs deriving from the serrated neoplasia pathway seem to be over-represented among interval carcinomas, making it even more important to early detect and resect SSLs [7].

The risk of iCRC increases when the polyp detection rate (PDR) and adenoma detection rate (ADR) are lower. An increased risk of iCRC has been reported if the PDR is < 20% [8]. The same is true for ADR, with an increased risk of iCRC when the ADR is < 20% [9]. Moreover, ADR has been shown to be inversely correlated with the incidence of CRC, with every 1% increase in ADR being associated with a 3% decrease in the risk of iCRC and a 5% decrease in the risk of fatal iCRC [10].

Human error is one of the factors responsible for a lower PDR. This error can (at least partly) be explained by so-called “inattention blindness.” This is a term used by psychologists to describe the lapse of focus, even when the motivation is strong to maintain concentrated [11, 12]. This issue has been addressed in several studies by including a second observer during colonoscopy which was shown to be beneficial for the detection of polyps, especially for the detection of diminutive polyps and adenomas [13–15].

Recently, computer-aided detection (CADe) systems have become increasingly popular as a new solution to the human error in detecting polyps. CADe systems use deep learning to improve polyp detection in a more consistent and reliable way than a second human observer can do. Over the past few years, several CADe systems have been developed and tested in real-time. Various meta-analyses, that included randomized controlled trials (RCTs) comparing CADe-assisted colonoscopy with conventional colonoscopy (CC), have shown a significant increase in ADR in favor of CADe-assisted colonoscopy (33.7–36.6% versus 22.9–26.6%, respectively). Similarly, PDR also significantly increased in the CADe group compared to the CC group (45.6% versus 30.6%, respectively) [16–18].

Recently, a novel CADe system (DISCOVERY; Pentax Medical, Tokyo, Japan) has been introduced. While pre-clinical studies have shown 90% sensitivity and 80% specificity with this CADe system [19], the system has not been systematically evaluated during real-time colonoscopy to date. The aim of this study was therefore to investigate the feasibility and safety of this CADe system during in vivo colonoscopy in three European tertiary referral centers.

## Methods

### Study design

In order to analyze the feasibility and safety of the novel CADe system, a prospective study including 90 colonoscopies was performed using this CADe system. All colonoscopies were performed in three European tertiary referral centers (Radboudumc, Nijmegen, the Netherlands; Universitätsklinikum Erlangen, Erlangen, Germany; and Vita-Salute San Raffaele University, Milan, Italy), with each center including 30 patients to ensure equal patient distribution.

### Outcomes

To study feasibility, inspection time, detection ratios (PDR, ADR, and SSL detection rate (SDR)), and number of false positives (as rated by the endoscopist) were taken as a composite endpoint. To study safety, (severe) adverse events ((S)AEs) were collected. Other outcomes included total number of polyps (PPC), adenomas (APC) and SSLs (SPC) per colonoscopy, Boston Bowel Preparation Score (BBPS), and total procedure time. Additionally, endoscopists were asked to subjectively evaluate the number of false negatives and user friendliness of the CADe system, rated on a scale from 1 (worst) to 10 (best).

### Participants

Patients aged ≥ 18 years, scheduled for diagnostic, non-immunochemical fecal occult blood test (iFOBT) based screening or surveillance colonoscopy were eligible to participate in the study. Patients were excluded if they were known with a colorectal tumor or with polyp(s) on referral, inadequately corrected anticoagulation disorder or continuous anticoagulation medication use during colonoscopy, American Society of Anesthesiologists (ASA) score ≥ 3, a known or suspected diagnosis of inflammatory bowel disease, if they were referred for a therapeutic procedure (i.e., endoscopic resection, intervention for lower gastro-intestinal bleeding), or if the colonoscopy procedure was incomplete (i.e., if the cecum was not reached or the procedure ended prematurely). Eligible patients were invited to participate in consecutive order, up to a maximum of 30 patients per study site (excluding screen failures). All participants gave written informed consent before participating in the study.

Patients who were found to have an inadequate bowel preparation, as measured by BBPS < 6, or a score of < 2 in any segment of the colon, were included in the analysis when the endoscopist decided to continue the colonoscopy. The reason was that the performance of the system in an inadequately prepared colon was also considered to be an outcome in this feasibility study.

### Materials and procedure

The CADe system uses a deep convolutional neural network (DCNN) to detect colorectal polyps. The DCNN has been developed with > 100,000 images from 788 polyps in 278 unique patients. Five expert endoscopists annotated the presence of polyps in this dataset. These annotations were considered the golden standard. For the training process of the DCNN, a dataset of 10,467 images from 504 different polyps was used. All images used for the training phase were provided by five centers from four countries. The ratio between inpatients and outpatients in the training dataset was 22% versus 78%. To evaluate the systems’ performance, videos rather than still images were used, to mimic the clinical setting as realistically as possible. For this, an independent test dataset was generated, consisting of 45 videos comprising of 15,534 single annotated video frames. To reduce the number of false positives, a minimum of three consecutive frames are considered positive before a detection signal (acoustically and/or visually) is provided. This results in a delay between the initial detection of a polyp and it being marked of ± 100 ms. For more information, we refer to Pfeifer et al., who described in great detail how this CADe system was developed [20].

**Fig. 1:**
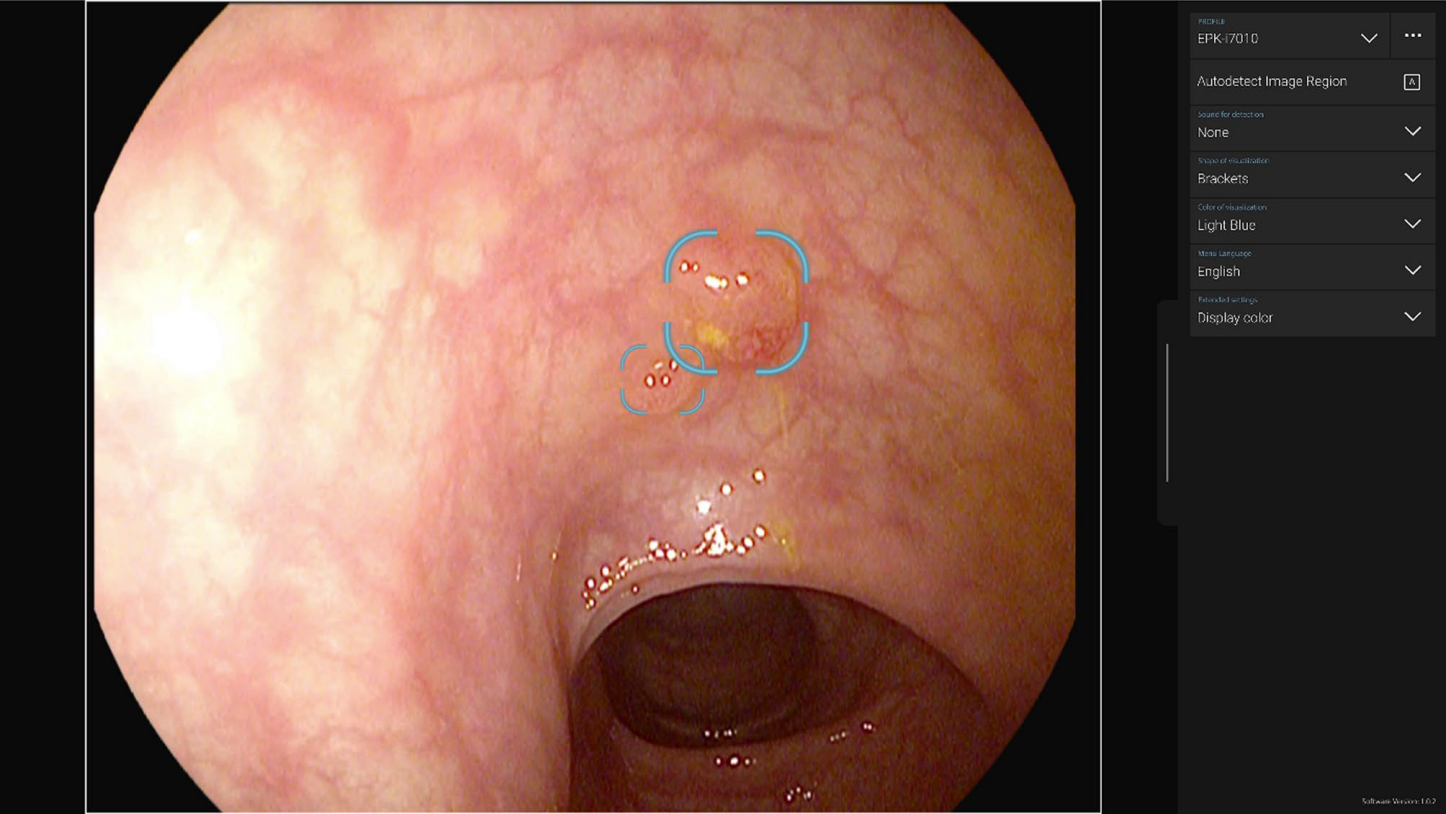
An example video output of a CADe system. Printed with permission of Pentax Medical EMEA

The CADe displays the video coming from the video processor with a delay of ± 15–30 ms, which is hardly noticeable to the human eye, on a second screen that is placed next to and parallel to the primary endoscopy monitor. Suspicious areas are highlighted by an overlaying bounding box on the endoscopic output video in real time (see Fig. 1 as an example). The system is intended to be used as a secondary monitor for the endoscopic system to assist the endoscopist in detecting colorectal polyps during colonoscopy. The diagnosis itself and the decision to perform treatment remains the responsibility of the endoscopist.

All colonoscopies were performed by experienced endoscopists (> 500 independently conducted colonoscopies). The studied CADe system was the first CADe system to be used for all participating endoscopists. All of them had the opportunity to practice with the system for a maximum of five colonoscopies before including patients in the study. To reduce operator-related variability, a maximum of three endoscopists per center participated in the study. All colonoscopies were performed using HD-WLE endoscopes (EC-38 i10 or EC-3890Fi, Pentax Medical). No complementary measures (i.e., cap devices) were used to increase polyp detection.

The CADe system was switched on during colonoscope withdrawal only. The endoscopist was instructed to look primarily on the main monitor during the procedure, and to look at the secondary screen only when the CADe system highlighted an area through an acoustic signal. Also, the endoscopist was instructed to aim for a withdrawal time of at least 6 min (excluding the time to perform polypectomy).

### Histopathological evaluation

All resected polyps were sent in separate containers for histopathological evaluation at the local center. All participating pathologists were trained as gastrointestinal pathologist.

### Data collection and management

The following outcome data were collected during each colonoscopy: number of resected polyps, procedure time, type of colonoscope, inspection time (excluding time for biopsies/polypectomies, as measured using a stopwatch), and BBPS. In addition, the following data were collected for each detected polyp: location, size, morphology according to the Paris classification [21], and the histopathological diagnosis. Other data that were collected included patients’ sex, age, smoking status, body mass index (BMI), family history of CRC, and the colonoscopy indication. To assess whether (S)AEs had occurred, patients were followed-up for a period of 30 days after the colonoscopy.

To evaluate the ease of use of the CADe, the performing endoscopists were asked to estimate the number of polyps that they considered to have missed without the CADe, the number of false negatives (i.e., polyps missed by the CADe system), and the number of false positives (i.e., acoustic notifications with no polyp present). For the latter, the colonoscopists were asked to categorize the number of false positives into five categories, i.e., 0–10, 10–20, 20–50, 50–100, and > 100. The user friendliness of each colonoscopy with the CADe system was rated on a scale from 1 (worst) to 10 (best).

All data were collected and stored anonymously in CastorEDC (Castor Electronic Data Capture, Ciwit BV, Amsterdam, the Netherlands), an online Electronic Data Capture platform.

### Ethical approval

The study protocol was approved by the local institutional review board in each participating center, and the study was performed in accordance with the Helsinki Declaration. The study was monitored in accordance with good clinical practice (GCP) principles. The study was registered in the Dutch Trial Register (https://trialsearch.who.int/Trial2.aspx?TrialID=NL8788).

### Statistical analysis

Categorical data are presented as counts and percentages. Continuous data are reported as mean (± standard deviation (SD)) or median (± interquartile range (IQR)) when appropriate. All analyses were performed using the software program IBM SPSS Statistics, version 25 (SPSS Inc., Chicago, IL, USA).

## Results

### Baseline characteristics

**Fig. 2:**
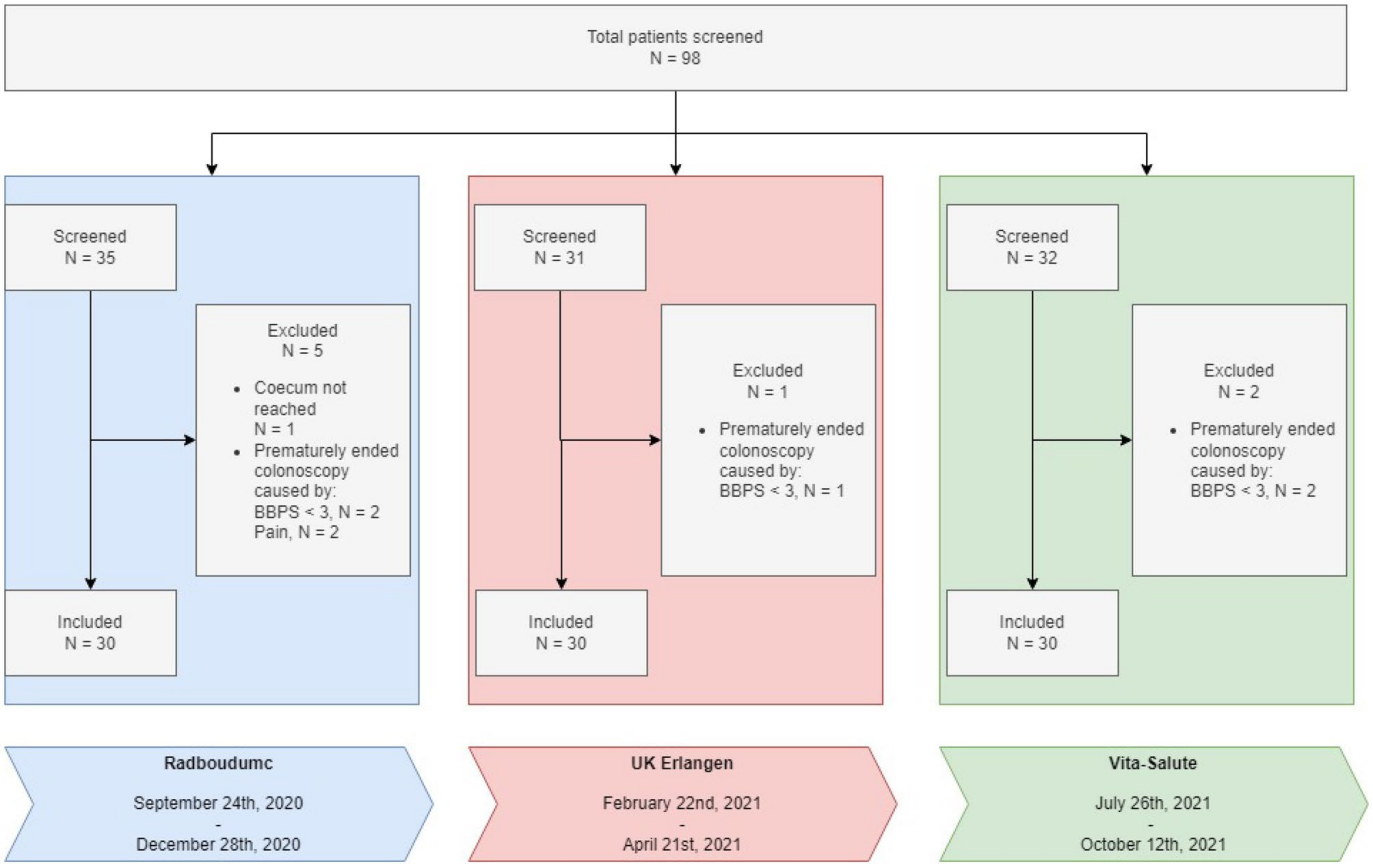
Flow diagram of inclusion

Ninety-eight patients gave their consent to participate in the study. Eight patients were excluded from analysis, because the colonoscopy was ended prematurely (n = 7) or the cecum was not reached (*n* = 1). Figure 2 represents the flow diagram of inclusion.

Baseline characteristics of the demographic and endoscopic data of all included patients are shown in Table 1. Of all 90 patients included, 30 (33.3%) were women. In addition, three patients with Lynch syndrome and one patient with serrated polyposis syndrome (SPS) were included. Two centers performed all colonoscopies under propofol as per local protocol, whereas in the other center, midazolam was used. The BBPS was rated as inadequate in 15/30 procedures in one center, and 1/30 in both other centers. The mean inspection time (excluding procedure time) was 10.8 ± 4.3 min. The median inspection time for the first five colonoscopies performed per endoscopist was 11.5 (IQR 5.00), which significantly decreased from the sixth colonoscopy onward to 9.00 (IQR 2.25, *p* < 0.001), data not shown.

**Table 1:** Baseline characteristics of demographic and endoscopic data. CADe computer aided detection system; y years; SD standard deviation; BMI body mass index; iFOBT immunochemical fecal occult blood test; BBPS Boston bowel preparation scale; ‡ missing for one case; † adequate BBPS score if sum ≥ 6 and every segment ≥ 2

**Variable**	**CADe colonoscopy, *n*** **= 90**
**Demographic data**	
Age (y), mean ± SD	62.3 ± 12.1
BMI, mean ± SD	25.9 ± 4.9
Sex (female), *n* (%)	30 (33.3)
Indication for colonoscopy, *n* (%) Diagnostic Screening (non-iFOBT) Surveillance	38 (42.2) 12 (13.3) 40 (44.4)
Hereditary cancer syndrome	4 (4.4)
Smoking, *n* (%)	14 (15.7)
Family history of CRC‡, *n* (%)	19 (21.3)
**Endoscopic data**	
Endoscope, *n* (%) EC38-i10 EC-3890Fi	80 (88.9) 10 (11.1)
General anesthesia (no), *n* (%)	59 (65.6)
Total BBPS score, mean ± SD	7.1 ± 1.9
BBPS rank adequate†, *n* (%)	73 (81.1)
Procedure time (min), mean ± SD	23.0 ± 9.3
Inspection time excluding biopsy/polypectomy time (min), mean ± SD	10.8 ± 4.3

In addition, Supplementary Table 1 shows the exact number of included colonoscopies per examinator. In one center (Universitätsklinikum Erlangen), all colonoscopies were performed by one endoscopist. In other centers, more than one endoscopist have performed the colonoscopies.

#### Polyp characteristics

Polyp characteristics are shown in Table 2. A total of 87 polyps were detected in 50 colonoscopies (55.5%). In 23/90 patients (25.6%), more than 1 polyp was detected. Forty-four polyps (50.6%) were resected in the right-sided (i.e., from cecum to splenic flexure) colon, while 43 polyps (49.4%) were resected in the left-sided colorectum. The majority of removed polyps was diminutive (*n* = 62, 71.3%) and histologically diagnosed as adenoma (*n* = 41, 47.1%). Twelve polyps (13.8%) were histologically diagnosed as SSL. Most SSLs (10/12, 83.3%) were found in the proximal colon, whereas most hyperplastic polyps (20/23, 87.0%) were found in the distal colorectum (see Supplementary Table 2 for polyp location per histopathological diagnosis). Also, five polyps were histologically diagnosed as inflammatory polyp, two as normal tissue, one as traditional serrated adenoma (TSA), and one as connective tissue polyp. Two polyps could not be retrieved.

**Table 2:** Polyp characteristics. CADe, computer-aided detection system; SSL, sessile serrated lesion; HP, hyperplastic polyp

**Variable**	**Polyps detected during CADe colonoscopy,** *n* **= 87**
Polyps removed per patient, *n* (%) 0 1 2 3 ≥ 4	40 (44.4) 27 (30.0) 16 (17.8) 3 (3.3) 4 (4.4)
Location, *n* (%) Cecum Ascending Transverse Descending Sigmoid Rectum	10 (11.5) 24 (27.6) 10 (11.5) 16 (18.4) 17 (19.5) 10 (11.5)
Size, *n* (%) 0–5 mm 6–10 mm 10–20 mm > 20 mm	62 (71.3) 17 (19.5) 7 (8.0) 1 (1.1)
Shape (Paris classification), *n* (%) Ip Is IIa IIb	- 43 (49.4) 35 (40.2) 9 (10.3)
PA-conclusion, *n* (%) Adenoma SSL HP Other	41 (47.1) 12 (13.8) 23 (26.4) 11 (12.6)

### Detection rates and median number of polyps, adenomas, and SSLs per colonoscopy

Detection rates for different types of polyps and median number of polyps, adenomas, and SSLs per colonoscopy are shown in Table 3. The PDR was 55.6%, ADR 28.9%, and SDR 11.1%. The PPC was 1 (IQR 2; mean 0.97 ± 1.19 (SD)), APC was 0 (IQR 1; mean 0.46 ± 0.88), and SPC was 0 (IQR 0; mean 0.13 ± 0.40).

**Table 3:** Detection rates and median number of detected polyps. CADe, computer-aided detection system; PDR, polyp detection ratio; ADR, adenoma detection ratio; SDR, sessile serrated lesion detection ratio; PPC, number of polyps per colonoscopy; APC, number of adenomas per colonoscopy; SPC, number of sessile serrated lesions per colonoscopy; SD, standard deviation

**Variable**	**CADe colonoscopy,** ***n*** **= 90**
PDR, *n* (%)	50 (55.6)
ADR, *n* (%)	26 (28.9)
SDR, *n* (%)	10 (11.1)
PPC Mean (SD)	0.97 (1.19)
APC Mean (SD)	0.46 (0.88)
SPC Mean (SD)	0.13 (0.40)

No statistically significant differences were found in detection ratios and in PPC, APC, and SPC when comparing patients with inadequate bowel preparation to patients with adequate bowel preparation (see Supplementary Table 3).

### Safety

Frequency and types of (S)AEs are shown in Table 4. In most (88/90, 97.8%) colonoscopies, no (S)AEs were observed. In one patient, an intraprocedural bleeding after removal of a diminutive lesion was successfully treated with a hemostatic clip. A second patient experienced intraprocedural bradycardia. After administration of anticholinerchic medication, the heart rate normalized, and the procedure could be completed. None of the AEs were considered to be related to the CADe system. No (S)AEs occurred within 30 days after the colonoscopy procedure.

**Table 4:** (Severe) adverse events

**Type of event**	**Number of events,** ***n*** **(%)**
Adverse event None Major bleeding Perforation Bradycardia	88 (97.8) 1 (1.1) - 1 (1.1)
Severe adverse event	-

### CADe usage

Table 5 shows the evaluation of the CADe system by the endoscopists. Endoscopists had not the impression that the CADe system had detected additional polyps (range 0–1), nor that the system had missed any of the polyps that were first detected by the endoscopist (range 0–1). During most colonoscopies, ≤ 20 false positives were registered (81/90, 90%). False positives were mostly caused by residual colonic fluid, colonic folds, and shadows (e.g., from colonic folds). Endoscopists rated the user friendliness of the CADe system with a median of 8/10 (IQR 2).

**Table 5:** Feasibility parameters of CADe system. CADe computer aided detection system; IQR interquartile range

**Feasibility parameter**	**Outcome**
Number of additional polyps discovered with CADe system, median (range)	0 (0–1)
Number of polyps missed with CADe system, median (range)	0 (0–1)
Frequency of false positives, *n* (%) 0–10 10–20 20–50	42 (46.7) 39 (43.3) 9 (10.0)
User friendliness, median (IQR)	8.0 (2)

## Discussion

In this multicenter feasibility and safety study investigating a novel CADe system, the mean inspection time was 10.8 ± 4.3 min, and PDR, ADR, and SDR were found to be 55.6%, 28.9%, and 11.1%, respectively. No (S)AEs related to the CADe system were observed during the 30-day post-colonoscopy study period. Users estimated < 20 false positives in most colonoscopies (81/90, 90%) and rated user friendliness of the CADe system with a median of 8/10.

Several other studies that included 184–502 subjects and investigated different CADe systems have reported CADe system assisted inspection times of 6.16–7.30 min [22–27]. The median inspection time of 10.8 min in our study suggests that there may be some room for improvement; however, when excluding the first five colonoscopies that had been performed by each participating endoscopist, the median inspection time in our study significantly reduced from 11.5 (IQR 5.00) to 9.00 (IQR 2.25, *p* < 0.001) minutes. This withdrawal time of 9.00 min in our study is comparable to the inspection time of 9.30 min reported in a smaller single-center study that included 83 subjects, which is however still approximately three times as many as the 30 subjects per study site in our study [28]. Therefore, we are optimistic that CADe-assisted inspection time will reduce to an inspection time of 6–8 min when endoscopists have become more experienced in using this CADe system.

Since 2019, seven RCTs, including > 5500 subjects, have shown increased detection rates of polyps, adenomas, and SSLs with CADe-assisted colonoscopy, although the findings were not always statistically significant [22–27, 29]. In RCTs that reported on PDR, this was found to significantly increase from 25.4–37.8% during CC to 38.3–56.0% during CADe-assisted colonoscopy [22–25, 27, 29]. In all but one RCTs, ADR significantly increased from 16.5–40.4% in CC to 28.9–54.8% in CADe assisted colonoscopy [22, 24–27, 29]. The only RCT that did not show a significant increase in ADR was powered to detect a 15% difference in AMR, rather than ADR [23]. Although two RCTs that reported on SDR found an increase from 0.3–5.2% for CC to 0.8–7.0% for CADe-assisted colonoscopy, neither of these were found to be statistically significant [25, 26]. The PDR in our study was higher than the PDRs reported in the CADe arms of almost all RCTs that reported on PDR [22, 24, 25, 27, 29]. Also, the SDR in our study was remarkably high, especially when compared to the reported SDRs in previous RCTs. In Table 6, an overview of study characteristics and outcomes of previous studies on various CADe systems including the current study is shown.

**Table 6:** Study characteristics and outcomes of other CADe system studies. RCT, randomized controlled trial; PDR, polyp detection rate; ADR, adenoma detection rate; SDR, sessile serrated lesion detection rate; NR, not reported; **‡**characteristics and outcomes of interventional (CADe) arm only; †screening/surveillance reported as one category; ¤0–5 mm and 6–10 mm reported as one category; ◊6–10 mm and > 10 mm reported as one category; *significant difference compared to control group

	**Liu et al.** [25] 2020	**Liu et al.** [27] 2020	**Repici et al.** [26] 2020	**Su et al.** [29] 2019	**Wang et al.** [22] 2019	**Wang et al.** [24] 2020	**Wang et al.** [23] 2020	**Current study**
**Country**	China	China	Italy	China	China	China	China	Germany, Italy, Netherlands
**Study design**	RCT	RCT	RCT	RCT	RCT	RCT	RCT, tandem	Prospective cohort
**Endoscopist experience**	All levels	NR	Experienced	Experienced	All levels	Experienced	Experienced	Expert
**Subjects (*n*) ‡**	393	508	341	308	522	484	184	90
**Indication (%)‡** Screening Surveillance Diagnostic	21.16 - 78.84	5.91 - 94.09	22.6 25.2 22.3	37.34† - 62.66	7.66 - 92.34	17 - 83	31.52 10.33 58.15	13.3 44.4 42.2
**Detection rates (%)‡** PDR ADR SDR	47.07* 29.01* 0.76	43.65* 39.10* NR	NR 54.8* 7.0	38.3* 28.9* NR	45.02* 29.12* NR	52* 34* NR	55.98* 34.78 NR	55.6 28.9 11.1
**Polyp size (%)‡** 0–5 mm 6–10 mm > 10 mm	77.66* 19.68 2.66	66.40* 25.20 8.40	92¤* 11	70.62* 29.38◊* -	80.12* 16.67* 3.21	81* 17 2	NR	71.3 19.5 9.1
**Polyp morphology (%)‡** Pedicle Sessile Flat Laterally spreading tumor	10.11 89.89* NR -	10.70 37.04* 52.36* NR	NR	10.17 42.37* 47.46* NR	9.84 35.34* 54.82* NR	5 94* NR 1	NR	- 49.4 50.5 NR

Regarding polyp size and morphology, our results are in line with previous studies. For example, in previous studies, 66.4–80.1% of the detected polyps in the interventional arm were ≤ 5 mm, which is consistent with our findings [22, 24, 25, 27, 29]. Additionally, across all RCTs, the vast majority of polyps (89–94%) were flat or had a sessile morphology, which is again consistent with the results of the current study, in which 100% of detected polyps were flat or sessile (i.e., Paris classification Is, IIa and IIb) [22, 24, 25, 27, 29].

Most detected SSLs (10/12, 83.3%) were found in the right-sided colon. Previous meta-analyses have shown that CADe system use resulted in an increased detection of flat and sessile polyps, right-sided polyps and SSLs, among some other characteristics [17, 30]. It is as yet unclear whether the high SDR in our study is caused by the typical morphology (i.e., flat) and location (i.e. right-sided colon) of SSLs, or whether the detection of SSLs irrespective of their endoscopic characteristics is increased by use of this CADe system [31, 32].

To our knowledge, we are the first to report quantitative data on the user experience of the investigated CADe system. According to the endoscopists’ experience, the CADe system did not result in the detection of additional polyps, nor did it miss polyps. User friendliness of the CADe system was reported as good with a median of 8.0, and all users reported the system as easy to use. Even though endoscopists noted less than 20 false positive alarms in the far majority of colonoscopies, this was indicated as a drawback of the system. However, this was thought to be caused by the frequent acoustic notifications when a suspicious area was highlighted, which was reported as “annoying” by 3/8 endoscopists that performed 48.9% of all colonoscopy procedures. Endoscopists were instructed to primarily look on the main monitor, and only to look on the secondary (CADe) monitor when an acoustic notification was heard. In many cases, by the time the endoscopist had changed his/her view to the secondary monitor, the suspected area was no longer highlighted. Also, in many cases, a particular “suspected” area was highlighted multiple times, leading to repeated acoustic notifications caused by the same area. It seems likely that when the CADe system is displayed on the primary monitor, false positive observations will no longer be experienced as annoying, because the endoscopist immediately sees why the system is alerting him/her, or, alternatively, because the acoustic notification is switched off by the endoscopist. Moreover, it is expected that the number of false positives will anyhow be reduced when the next update of the CADe system will be launched by early 2022.

The expected shorter inspection time with increasing CADe system experience, the encouraging detection rates, the relatively low incidence of false positives, and the absence of (S)AEs make the implementation of this CADe system in real-time colonoscopy practice feasible and safe. Moreover, both the PDR and SDR of the current study were rather high compared to previous literature with other CADe systems. Although we are optimistic about these detection rates, future well-designed randomized trials powered to detect a clinically and statistically significant improvement in the quality indicator ADR are needed. As 15–20% of CRCs develop from the serrated pathway and SSLs seem to be over-represented among iCRCs, it will be of high interest to evaluate SDR as additional outcome [7]. Finally, future studies should also focus on the detection of lesions that are easily overlooked without an CADe system, such as small (< 10 mm) and nonpolypoid lesions [23].

Our study has several strengths. First, it is the first international study investigating a novel CADe system in a multicenter setting. Most previous studies have been performed in Asia as single-center studies. Only one previous study was performed as multicenter study, with different participating centers from Italy. Second, the study was performed in real-time colonoscopy practice. Third, this is the first study to quantitatively present data on the safety and user experience of a CADe system.

Some limitations of this study also need to be acknowledged. First, our study was intended to be explorative, and therefore, no control group was included. This makes it difficult to determine the objective additional role of this CADe system. Second, the definition we used for a false positive (i.e., acoustic notifications with no polyp present) may have resulted in a relatively high number of false positives which complicated the counting of them. Currently, no standardized definition of false positives or negatives is available, making it hard to compare various CADe validation studies [33]. This is illustrated by the fact that in both RCTs published by Wang et al., false positives were defined as a “detected lesion which was continuously traced by the system.” This resulted in a rather low false positive rate (0.075–0.1) per colonoscopy [22, 24]. In contrast, in a frame-by-frame analysis by Brand et al., false positives were defined as any frame that included a “detected area that was not in contact with a polyp.” This more strict definition resulted in a rather high number of false positives per colonoscopy (mean number per of false positives colonoscopy: 101) [34]. Nevertheless, our definition is in line with the proposed definition by Bernal et al. at the Medical Image Computing and Computer Assisted Intervention (MICCAI) conference [35]. Although we were unable to count the number of false positives, our results are in line with a post hoc analysis by Hassan et al., who reported a mean number of false positives of 27.3 ± 13.1 per colonoscopy when using a similar definition [36]. Lastly, bowel preparation was inadequate in 17/90 (18.9%) colonoscopies and in even 15/30 (50.0%) colonoscopies in one participating center. Although endoscopists rated bowel preparation as sufficient to proceed with the colonoscopy, this may have impeded polyp detection and removal on the one hand, but also may have led to increased false positive rates on the other hand.

In conclusion, the use of this CADe system in real-time colonoscopy practice is feasible and safe. Although PDR and SDR were found to be high compared to previous studies investigating other CADe systems, future studies are needed to confirm these increased detection rates. These studies should preferably be well-designed RCTs and should among other endpoints focus on improving ADR, which is until now the most widely accepted colonoscopy quality indicator.

## Supplementary Information

Below is the link to the electronic supplementary material.

Supplementary file1 (DOCX 14 KB)

Supplementary file2 (DOCX 14 KB)

Supplementary file3 (DOCX 16 KB)

## Data Availability

The datasets generated during and/or analyzed during the current study are available from the corresponding author on reasonable request.
