# Supplementary material for: Real-time colorectal polyp detection using a novel computer-aided detection system (CADe): a feasibility study
Source: Int J Colorectal Dis. 2022 Sep 27;37(10):2219–28. doi: 10.1007/s00384-022-04258-9 (PMC9560918; doi:10.1007/s00384-022-04258-9)
Supplement: Supplementary file 1 — Supplementary file1 (DOCX 14 KB) [file 384_2022_4258_MOESM1_ESM.docx]

|  | **Colonoscopies, n (%)** |
| --- | --- |
| **Radboudumc**  Prof. Siersema  Dr. van Dop  Dr. Hazewinkel | 2 (6.7)  17 (56.7)  11 (36.7) |
| **U.K. Erlangen**  Prof. Rath | 30 (100) |
| **Vita Salute**  Prof. Testoni  Dr. Esposito  Dr. Viale | 4 (13.3)  16 (53.3)  10 (33.3) |

**Supplementary table 1** Distribution of colonoscopies per investigator per participating center.
