# Supplementary material for: Real-time colorectal polyp detection using a novel computer-aided detection system (CADe): a feasibility study
Source: Int J Colorectal Dis. 2022 Sep 27;37(10):2219–28. doi: 10.1007/s00384-022-04258-9 (PMC9560918; doi:10.1007/s00384-022-04258-9)
Supplement: Supplementary file 2 — Supplementary file2 (DOCX 14 KB) [file 384_2022_4258_MOESM2_ESM.docx]

|  | Adenoma | HP | SSL | Other |
| --- | --- | --- | --- | --- |
| Cecum, n (%) | 4 (9.8) | - | 4 (33.3) | 2 (18.2) |
| Ascending colon, n (%) | 16 (39.0) | 1 (4.3) | 4 (33.3) | 3 (27.3) |
| Transverse colon, n (%) | 5 (12.2) | 2 (8.7) | 2 (16.7) | 1 (9.1) |
| Descending colon, n (%) | 6 (14.6) | 6 (26.1) | 1 (8.3) | 3 (27.3) |
| Sigmoid, n (%) | 8 (19.5) | 7 (30.4) | 1 (8.3) | 1 (9.1) |
| Rectum, n (%) | 2 (4.9) | 7 (30.4) | - | 1 (9.1) |

**Supplementary Table 1** Location per polyp diagnosis. HP hyperplastic polyp; SSL sessile serrated lesion. Other: inflammatory polyp (n = 5), normal tissue (n = 2), traditional serrated adenoma (n = 1), connective tissue polyp (n = 1), not retrieved during colonoscopy (n = 2).
