# Supplementary material for: Real-time colorectal polyp detection using a novel computer-aided detection system (CADe): a feasibility study
Source: Int J Colorectal Dis. 2022 Sep 27;37(10):2219–28. doi: 10.1007/s00384-022-04258-9 (PMC9560918; doi:10.1007/s00384-022-04258-9)
Supplement: Supplementary file 3 — Supplementary file3 (DOCX 16 KB) [file 384_2022_4258_MOESM3_ESM.docx]

| **Variable** | **Adequate BBPS**  **n=73** | **Inadequate BBPS**  **N=17** | **P-value** |
| --- | --- | --- | --- |
| PDR, n (%) | 39 (53.4) | 11 (64.7) | 0.399 |
| ADR, n (%) | 22 (30.1) | 4 (23.5) | 0.588 |
| SDR, n (%) | 8 (11.0) | 2 (11.8) | 0.924 |
| PPC, median (IQR) | 1 (2) | 1 (2) | 0.688 |
| APC, median (IQR) | 0 (1) | 0 (1) | 0.445 |
| SPC, median (IQR) | 0 (0) | 0 (0) | 0.880 |

**Supplementary table 2** Detection rates for adequate and inadequate bowel preparation. BBPS Boston Bowel Preparation Score; PDR polyp detection ratio; ADR adenoma detection ratio; SDR sessile serrated lesion detection ratio.
